# Spatiotemporal binding of cyclophilin A and CPSF6 to capsid regulates HIV-1 nuclear entry and integration

**DOI:** 10.1128/mbio.00169-25

**Published:** 2025-02-27

**Authors:** Zachary Ingram, Christopher Kline, Alexandra K. Hughson, Parmit K. Singh, Hannah L. Fischer, Rajalingham Radhakrishnan, Gregory A. Sowd, Nayara F. B. Dos Santos, Barbie K. Ganser-Pornillos, Simon C. Watkins, Melissa Kane, Alan N. Engelman, Zandrea Ambrose

**Affiliations:** 1 Department of Microbiology and Molecular Genetics, Division of Infectious Diseases, University of Pittsburgh School of Medicine, Pittsburgh, Pennsylvania, USA; 2 Pittsburgh Center for HIV Protein Interactions, Division of Infectious Diseases, University of Pittsburgh School of Medicine, Pittsburgh, Pennsylvania, USA; 3 Department of Infectious Diseases and Microbiology, University of Pittsburgh School of Public Health, Pittsburgh, Pennsylvania, USA; 4 Department of Cancer Immunology and Virology, Dana-Farber Cancer Institute, Boston, Massachusetts, USA; 5 Department of Medicine, Harvard Medical School, Boston, Massachusetts, USA; 6 Department of Biochemistry, University of Utah, Salt Lake City, Utah, USA; 7 Department of Cell Biology, University of Pittsburgh School of Medicine, Pittsburgh, Pennsylvania, USA; 8 Department of Pediatrics, Division of Infectious Diseases, University of Pittsburgh School of Medicine, Pittsburgh, Pennsylvania, USA; Columbia University Medical Center, New York, New York, USA

**Keywords:** human immunodeficiency virus, capsid, host-pathogen interactions, CPSF6, cyclophilin A, lenacapavir

## Abstract

Human immunodeficiency virus type 1 (HIV-1) capsid, which is the target of the antiviral lenacapavir, protects the viral genome and binds multiple host proteins to influence intracellular trafficking, nuclear import, and integration. Previously, we showed that capsid binding to cleavage and polyadenylation specificity factor 6 (CPSF6) in the cytoplasm is competitively inhibited by cyclophilin A (CypA) binding and regulates capsid trafficking, nuclear import, and infection. Here, we determined that a capsid mutant with increased CypA binding affinity had significantly reduced nuclear entry and mislocalized integration. However, disruption of CypA binding to the mutant capsid restored nuclear entry, integration, and infection in a CPSF6-dependent manner. Furthermore, relocalization of CypA expression from the cell cytoplasm to the nucleus failed to restore mutant HIV-1 infection. Our results clarify that sequential binding of CypA and CPSF6 to HIV-1 capsid is required for optimal nuclear entry and integration targeting, providing insights for the development of antiretroviral therapies, such as lenacapavir.

**IMPORTANCE:**

Human immunodeficiency virus (HIV) encodes a protein that forms a conical shell, called a capsid, that surrounds its genome. The capsid has been shown to protect the viral genome from innate immune sensors in the cell, to help transport the genome toward and into the nucleus, to keep the components of reverse transcription together for conversion of the RNA genome into DNA, and to target viral DNA integration into specific regions of the host genome. In this study, we show that HIV hijacks two host proteins to bind to capsid sequentially in order to choreograph the precise order and timing of these virus replication steps. Disruption of binding of these proteins to capsid or their location in the cell leads to defective HIV nuclear import, integration, and infection. Mutations that exist in the capsid protein of HIV in infected individuals may reduce the efficacy of antiretroviral drugs that target capsid.

## INTRODUCTION

Human immunodeficiency virus type 1 (HIV-1) capsid is a unique structure formed from capsid protein (CA) monomers that assemble into approximately 250 hexamers and precisely 12 pentamers (1, 2). Following HIV-1 fusion with the target cell membrane, the capsid enters the cytoplasm where it protects the viral RNA from recognition by host innate immune factors (3). Simultaneously, the HIV-1 capsid surface is an interface for binding host motor proteins and their adapters to promote microtubule trafficking (4–6). At the nucleus, specific host nucleoporin proteins bind to HIV-1 capsid to facilitate nuclear import of the HIV-1 genome (7–12).

Throughout these post-entry steps, HIV-1 capsid undergoes a poorly defined disassembly process, termed capsid uncoating, which is affected by several host proteins (13, 14). Capsid uncoating likely occurs at the nuclear pore complex (NPC) or within the nucleus, allowing completion of reverse transcription of the viral genome into double-stranded DNA, which is integrated into host chromatin (15–19). As HIV-1 capsid does not have a known cellular structural counterpart and is involved in numerous post-entry steps, it is a useful target for antiretroviral therapeutics. Lenacapavir (LEN), the first antiretroviral inhibitor that targets HIV-1 capsid, has been approved for use in humans (20, 21). Interestingly, LEN inhibits multiple steps of HIV-1 replication (20, 22).

Optimal HIV-1 replication depends on capsid binding to multiple host proteins. Cyclophilin A (CypA) is a cellular peptidyl prolyl isomerase involved in protein folding and trafficking (23–25) and binds to HIV-1 CA (26, 27). CypA promotes early HIV-1 replication in a cell type-dependent manner that correlates with events prior to nuclear import (28–30). CypA binding to HIV-1 capsid additionally prevents binding of the restriction factor TRIM5α to capsid in human CD4+ T cells and macrophages (31, 32). TRIM5α is an antiretroviral restriction factor that oligomerizes on the HIV-1 capsid surface, forming hexagonal nets that destabilize capsid and impair HIV-1 replication (33–35). Beyond establishing a mechanism for how CypA promotes HIV-1 replication, these findings suggest that host factors compete for binding to the HIV-1 capsid surface and can alter replication outcomes.

Previously, we showed that CypA binding limits HIV-1 capsid binding to the host protein cleavage and polyadenylation specificity factor 6 (CPSF6) in the cytoplasm, impacting capsid uncoating, nuclear trafficking, and infection (36, 37). CPSF6 is localized to the nucleus where it functions as a pre-mRNA alternate polyadenylation factor (38). CPSF6 directly binds to HIV-1 capsid within a hydrophobic pocket between adjacent CA monomers (39–41), which is also the binding site of LEN (22). CPSF6 promotes capsid disassembly, nuclear import, and post-import translocation to nuclear speckles for HIV-1 DNA integration within gene-dense chromatin and speckle-associated domains (SPADs) (37, 42, 43).

Research on individual host protein interactions with HIV-1 capsid has elucidated key replication events. Here, we have explored the spatiotemporal kinetics of CypA and CPSF6 binding to capsid and the impact these interactions have on HIV-1 replication, using both CA mutants and small molecule inhibitors. The unique mutant HIV-1_AC-1_ was previously shown to have increased CypA binding affinity, resulting in failed nuclear import unless CypA binding to capsid was inhibited (44, 45). Introduction of the CA N74D mutation, which abolishes CPSF6 binding (40), reversed the replication defect of HIV-1_AC-1_ by an undetermined mechanism (45). Given an incomplete understanding of HIV-1_AC-1_ restriction and the relationship of CypA and CPSF6 binding to capsid, we hypothesized that increased CypA binding affinity to capsid prevents CPSF6 from accessing the HIV-1 capsid, leading to reduced nuclear import, integration, and infection. Furthermore, loss of CypA binding would restore HIV-1_AC-1_ capsid binding to CPSF6 and infection, suggesting that sequential binding of host factors to capsid is required for replication.

## RESULTS

### HIV-1_AC-1_ binds to TRIMCyp with higher affinity compared to wild-type (WT) HIV-1

**Fig 1:**
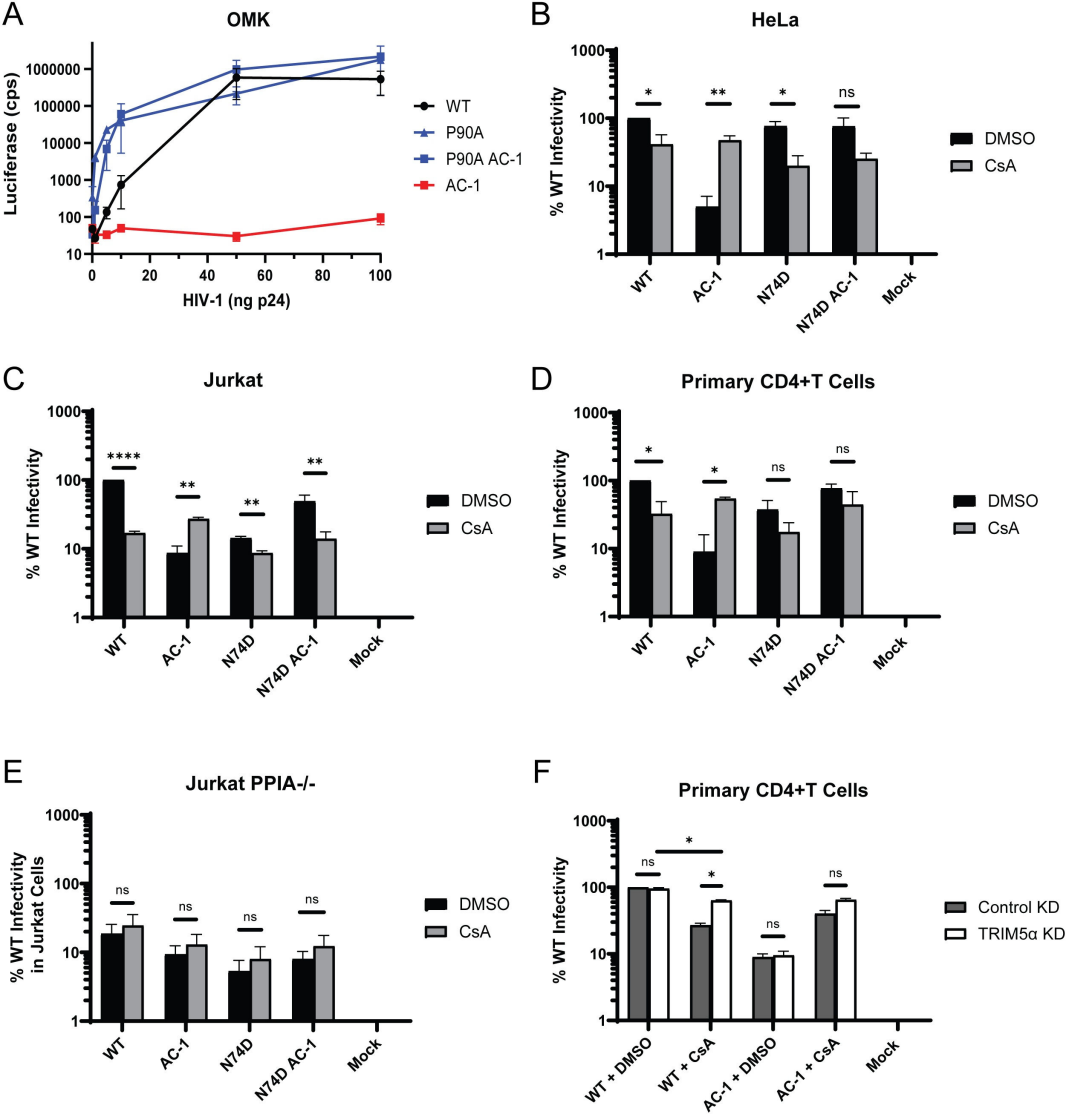
Increased CypA binding to HIV-1 capsid inhibits infection in a CPSF6-dependent manner. (**A**) OMK cells were infected with different amounts of WT HIV-1 and CA mutants (0.1–100 ng p24). Average luciferase values are shown for two independent experiments. (**B–E**) Infectivity of WT and mutant HIV-1 (10 ng p24) was determined after 48 h by luciferase activity in DMSO or 5–10 μM CsA in HeLa (*n* = 3) (**B**), Jurkat (*n* = 3) (**C**), primary CD4+ T cells (*n* = 2) (**D**), and Jurkat *PPIA^−/^*^−^ (*n* = 3) (**E**). (**F**) Primary CD4+ T cells were transduced with lentiviruses expressing control or TRIM5α miRNA prior to infection with WT and mutant HIV-1 (10 ng p24) in DMSO or 10 μM CsA. Infections were determined after 48 h by luciferase activity (*n* = 2). Error bars represent standard errors of the mean (SEM). Comparisons between infection conditions were analyzed by unpaired *t* tests. *P* values of <0.05 were considered significant and significant values are denoted as *, *P* < 0.05; **, *P* < 0.01; ***, *P* < 0.001; and ****, *P* < 0.0001. ns, *P* > 0.05.

HIV-1_AC-1_ was engineered by introduction of five amino acid substitutions within the CypA binding loop of CA (Fig. S1A). These substitutions were previously shown to increase CypA binding to purified CA N-terminal domain (NTD) protein approximately 1.5-fold, accompanied by comparatively high standard deviations from replicate microscale thermophoresis (MST) measures (45). We accordingly first strove to repeat binding assays in the presence of the mature capsid lattice, which is arguably more physiologically relevant than isolated NTD proteins. First, we built recombinant capsid-like particles (CLPs) and nanotubes from purified WT and AC-1 CA. Because the comparatively large sizes of these structures preclude the use of MST, we performed co-pelleting assays with purified CypA protein. These experiments failed to recapitulate higher binding affinity of CypA to AC-1 capsid substrates compared to WT (Fig. S1B through E). We next infected owl monkey kidney (OMK) cells, which express a natural fusion of TRIM5α and CypA, TRIMCyp, leading to restriction of HIV-1 infection via CypA binding to capsid (46, 47). As shown previously, TRIMCyp restriction of WT HIV-1 was overcome with increasing multiplicity of infection, which increases the amounts of capsid entering the cells (Fig. 1A). In contrast, increasing amounts of capsid failed to overcome the restriction of HIV-1_AC-1_ infection in OMK cells. Addition of the P90A CA mutation, which prevents CypA binding to HIV-1 capsid (48), alone or with the AC-1 mutations, alleviated restriction. These results are fully consistent with the notion that the CypA domain of TRIMCyp binds to HIV-1_AC-1_ with higher affinity than WT HIV-1 in the context of infected cells.

### Increased CypA binding inhibits HIV-1 infection in a CPSF6-dependent manner

Infectivity of HIV-1_AC-1_ was rescued to near WT levels by treating the cells with cyclosporine A (CsA), a calcineurin inhibitor that prevents CypA from binding to HIV-1 capsid, or by reducing CypA expression in target cells (45). In agreement with the literature, HIV-1_AC-1_ infection was significantly restricted in HeLa, Jurkat, and primary CD4+ T cells (Fig. 1B through D) and these defects were rescued to WT levels by CsA treatment. We compared WT HIV-1 and HIV-1_AC-1_ infectivity in Jurkat cells lacking the *PPIA* gene, which encodes CypA (30). In *PPIA^−/^*^−^ cells, WT HIV-1 and HIV-1_AC-1_ supported similar levels of infection with or without CsA treatment (Fig. 1E). Importantly, HIV-1_AC-1_ had similar infectivity as WT HIV-1 in *PPIA^−/^*^−^ cells, confirming that CypA binding leads to HIV-1_AC-1_ restriction. This was further demonstrated by addition of the P90A CA mutation in HIV-1_AC-1_, resulting in infectivity comparable to P90A HIV-1 (Fig. S2A).

The CA N74D substitution abolishes HIV-1 capsid binding to CPSF6 but maintains CypA binding (40, 49), and the N74D change was previously shown to counteract HIV-1_AC-1_ restriction (45). Indeed, we observed no difference in infection of N74D HIV-1 and N74D HIV-1_AC-1_ in multiple cell types (Fig. 1B through E). These results suggested that CPSF6 binding also contributes to HIV-1_AC-1_ restriction.

As previous work suggested that CypA binding to HIV-1 capsid shields it from TRIM5α binding in primary cells (31, 32), we hypothesized that HIV-1_AC-1_ also would be resistant to TRIM5α restriction. TRIM5α knockdown (KD) was performed in CD4+ T cells and confirmed by infection with N-tropic murine leukemia virus (N-MLV), which is restricted by TRIM5α (50, 51) (Fig. S2B). As previously reported, WT HIV-1 infection was inhibited by CsA treatment, which was partially restored by TRIM5α KD (Fig. 1F). As expected, WT and HIV-1_AC-1_ infected these cells similarly in the presence of CsA. However, HIV-1_AC-1_ infectivity was not significantly increased by TRIM5α KD in the presence of CsA. Thus, CypA binding to HIV-1_AC-1_ capsid prevents TRIM5α restriction like WT capsid.

### Increased CypA binding does not affect inherent HIV-1 capsid stability

**Fig 2:**
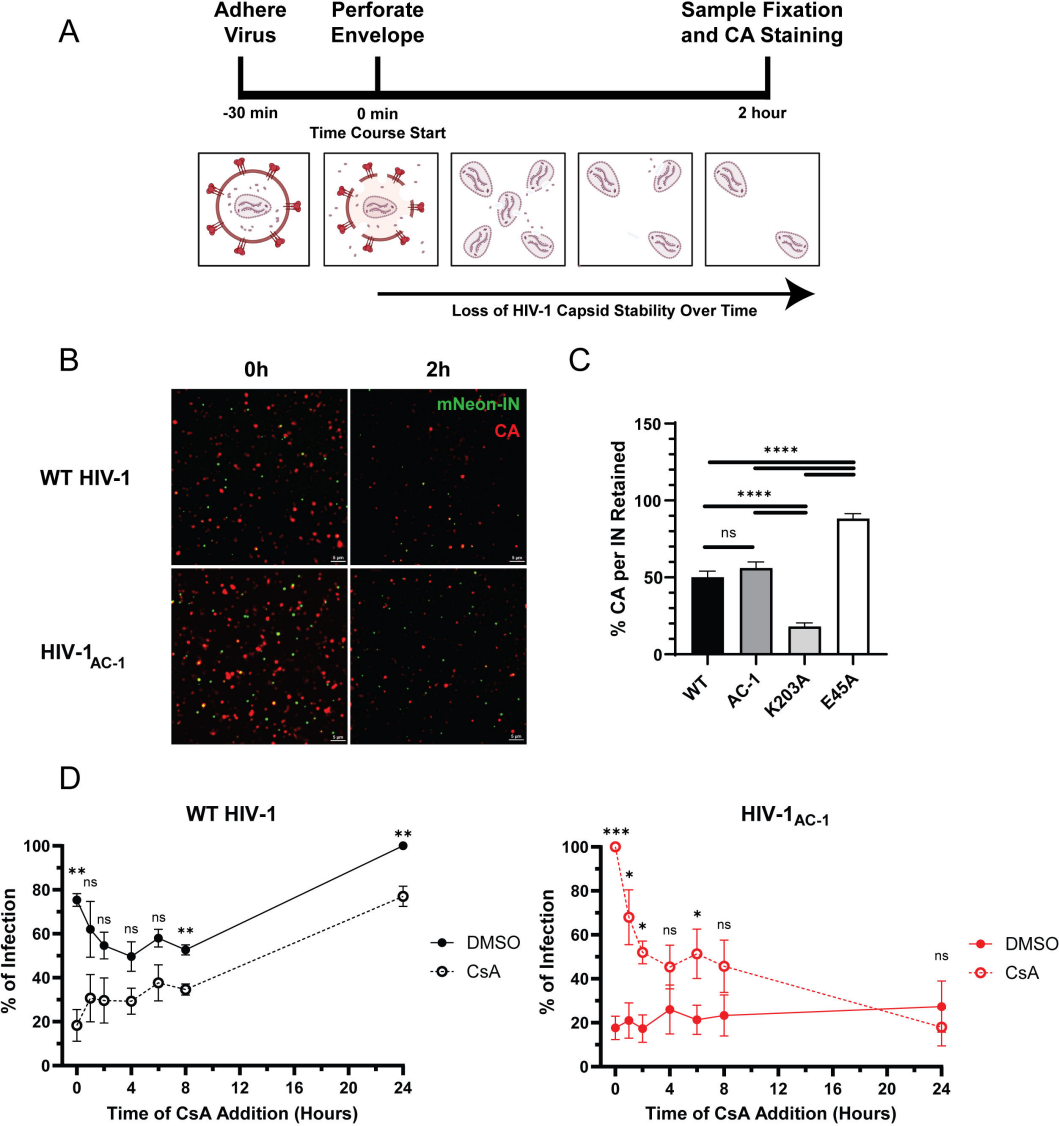
HIV-1_AC-1_ has similar capsid stability as WT HIV-1. (**A**) Schematic of the HIV-1 CA retention assay. (**B**) Representative TIRF images of HIV-1 samples fixed at 0 h and 2 h are shown. Scale bars denote 5 μm. (**C**) Quantification of the average HIV-1 CA retention normalized to mRuby3-IN for WT HIV-1 and HIV-1_AC-1_ (*n* = 3, eight fields/virus). (**D**) DMSO or 10 μM CsA was added at different time points to HeLa cells synchronously infected with WT HIV-1 or HIV-1_AC-1_ (*n* = 3). Infectivity was normalized to maximal infection (24 h in DMSO for WT and 0 h in CsA for AC-1). Error bars represent SEM and unpaired *t* tests were performed for comparisons between viruses (**C**) or conditions at each time point (**D**). *P* values <0.05 were considered significant and significant values are denoted as *, *P* < 0.05; **, *P* < 0.01; ***, *P* < 0.001; and ****, *P* < 0.0001. ns, *P* > 0.05.

To characterize the relative stabilities of WT HIV-1 and HIV-1_AC-1_ capsids, we used the *in vitro* CA retention assay (52), in which fluorescently labeled virions were stained for CA and imaged by microscopy (Fig. 2A). CA retention of HIV-1_AC-1_ was comparable to WT HIV-1 CA (~50% of initial CA signal), suggesting that their inherent stabilities were similar (Fig. 2B and C). The HIV-1 CA mutants K203A and E45A, which display rapid loss or delayed retention of CA, respectively, were included as controls (52). As expected, K203A capsids showed a significant decrease in retained CA (<20%), whereas E45A capsids maintained nearly 90% of CA (Fig. 2C).

Next, we investigated capsid stability in cells and whether binding to CypA leads to irreversible restriction of infection. HeLa cells were synchronously infected and CsA was introduced at different time points to disrupt CypA binding. Infection was measured 48 h later to assess at which time points CsA could still rescue infectivity (Fig. 2D; Fig. S2C and D). CsA addition at the onset of WT HIV-1 infection (0 h) resulted in a significant drop in infectivity likely due to CypA enhancing early replication events (29). In contrast, HIV-1_AC-1_ infectivity was maximal when CsA was introduced at 0 h and then decreased over time. However, HIV-1_AC-1_ infection was partially rescued by CsA up to 6 h, after which the addition of CsA did not significantly restore infectivity, indicating that HIV-1_AC-1_ capsid was not immediately destabilized by CypA binding but exhibited an irreversible change by 6 h post-infection.

### Increased CypA binding to HIV-1 capsid leads to failed nuclear import

**Fig 3:**
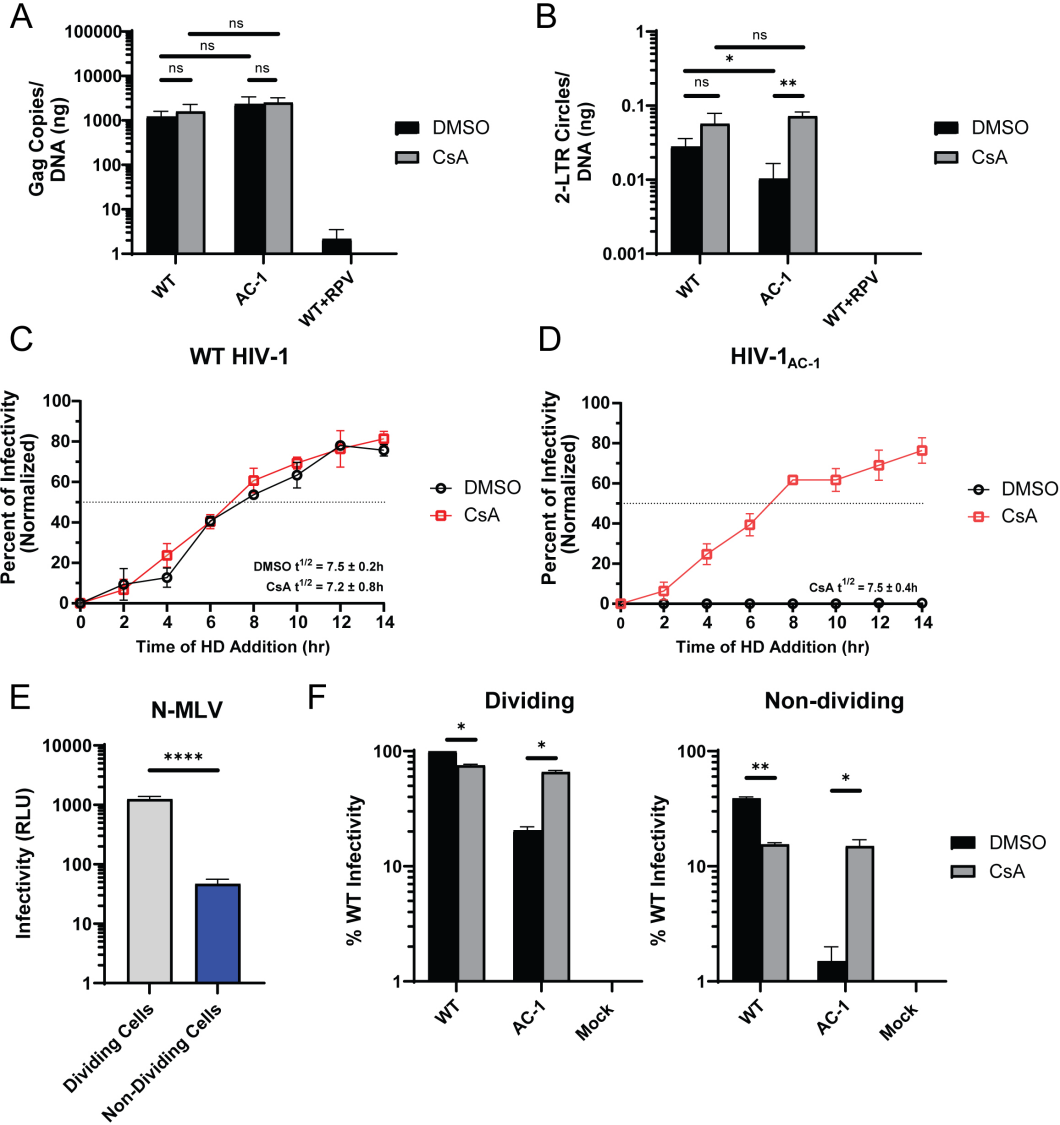
Increased CypA binding restricts HIV-1 nuclear import. (**A**) Reverse transcripts and (**B**) 2-LTR circles were measured by qPCR for WT HIV-1 and HIV-1_AC-1_ infection of HeLa cells treated with DMSO or 10 μM CsA after 24 h (*n* = 3). (**C and D**) Nuclear import kinetics were determined for WT HIV-1 (**C**) and HIV-1_AC-1_ (**D**) infection of HeLa cells in DMSO or 10 μM CsA containing media at different time points (*n* = 3). Infectivity was determined by luciferase activity and normalized to luciferase expression at 24 h post-infection. Horizontal dotted lines intersect the *y*-axis at 50% infectivity. (**E and F**) HeLa cells treated with or without aphidicolin were infected with N-MLV encoding luciferase (**E**) or WT HIV-1 and HIV-1_AC-1_ encoding mNeonGreen (**F**) in the presence of DMSO or 10 μM CsA (*n* = 2). Infectivity was measured by reporter gene expression at 48 h. Error bars represent SEM. Comparisons between infection conditions were analyzed by unpaired *t* tests. *P* values <0.05 were considered significant and significant values are denoted as *, *P* < 0.05; **, *P* < 0.01; and ****, *P* < 0.0001. ns, *P* > 0.05.

As HIV-1_AC-1_ capsid stability was comparable to WT HIV-1, we next investigated nuclear import (45). To determine if CypA binding to HIV-1_AC-1_ impacted reverse transcription, we quantified WT HIV-1 and HIV-1_AC-1_ *gag* DNA copies 24 h post-infection (Fig. 3A). HIV-1_AC-1_ and WT HIV-1 synthesized similar numbers of reverse transcripts, regardless of CsA treatment, suggesting that reverse transcription was not impaired. Nuclear import was measured by 2-long terminal repeat (2-LTR)-containing circle detection, which are formed by non-homologous end joining of unintegrated HIV-1 DNA in the nucleus (53). HIV-1_AC-1_ infection led to lower levels of 2-LTR circles, which were restored to WT HIV-1 levels when the cells were treated with CsA (Fig. 3B).

As 2-LTR circles form after nuclear import and require the completion of reverse transcription, their detection may not accurately reflect nuclear import kinetics. Thus, we performed the nuclear import kinetics (NIK) assay whereby cellular expression of a Nup62-GFP fusion containing a drug-inducible dimerizing domain leads to blockage of the NPC in the presence of a homodimerization (HD) drug, which prevents nuclear import of HIV-1 genomes (17). The assay was performed in cells infected with WT HIV-1 or HIV-1_AC-1_ with or without CsA. WT HIV-1 infection half-life (t_1/2_) was calculated as 7.5 ± 0.2 h post-infection without CsA and 7.1 ± 0.8 h with CsA (Fig. 3C; Fig. S3A), which was consistent with a previous report (17). In contrast, HIV-1_AC-1_ nuclear import was undetectable in the presence of dimethyl sulfoxide (DMSO), further indicating that this mutant is restricted at nuclear import (Fig. 3D; Fig. S3B). However, CsA rescued HIV-1_AC-1_ nuclear import with a t_1/2_ of 7.5 ± 0.4 h, suggesting that HIV-1_AC-1_ nuclear import proceeds similarly to that of WT HIV-1 when CypA binding is disrupted.

As the HIV-1 genome enters the nucleus both through the NPC and breakdown of the nuclear envelope during mitosis, restriction of HIV-1_AC-1_ infection may be enhanced in nondividing cells. HeLa cells were infected with viruses in the presence or absence of aphidicolin (54). N-MLV, which cannot infect nondividing cells (55, 56), served as a control for aphidicolin treatment (Fig. 3E). Infection of WT HIV-1 was reduced in nondividing cells regardless of CsA treatment, but HIV-1_AC-1_ infectivity was more dramatically reduced in nondividing cells without CsA (Fig. 3F). Addition of CsA rescued HIV-1_AC-1_ infectivity to WT HIV-1 + CsA levels, suggesting that HIV-1_AC-1_ uses NPCs in nondividing cells when CypA binding is inhibited.

We next investigated the contribution of the CypA homology domain (CHD) of NUP358 to nuclear import. NUP358 is a cytoplasmic ring nucleoporin, and NUP358 depletion inhibits HIV-1 nuclear import and infection (7, 57–61). While one group showed that the NUP358 CHD binds to the CypA binding loop of HIV-1 capsid to promote nuclear import (59, 61), another report suggested that it is not required for infection (62). To investigate the role of the NUP358 CHD in HIV-1_AC-1_ infection, we infected HT1080 cells edited with CRISPR/Cas9 to remove the CHD of NUP358 (NUP358delCyp) (63). No difference was observed for HIV-1_AC-1_ infection of delCyp or control cells with or without CsA treatment (Fig. S4), suggesting that interactions with NUP358 CHD are not responsible for restriction of HIV-1_AC-1_.

### Cytoplasmic but not nuclear CypA regulates HIV-1 nuclear import and infection

**Fig 4:**
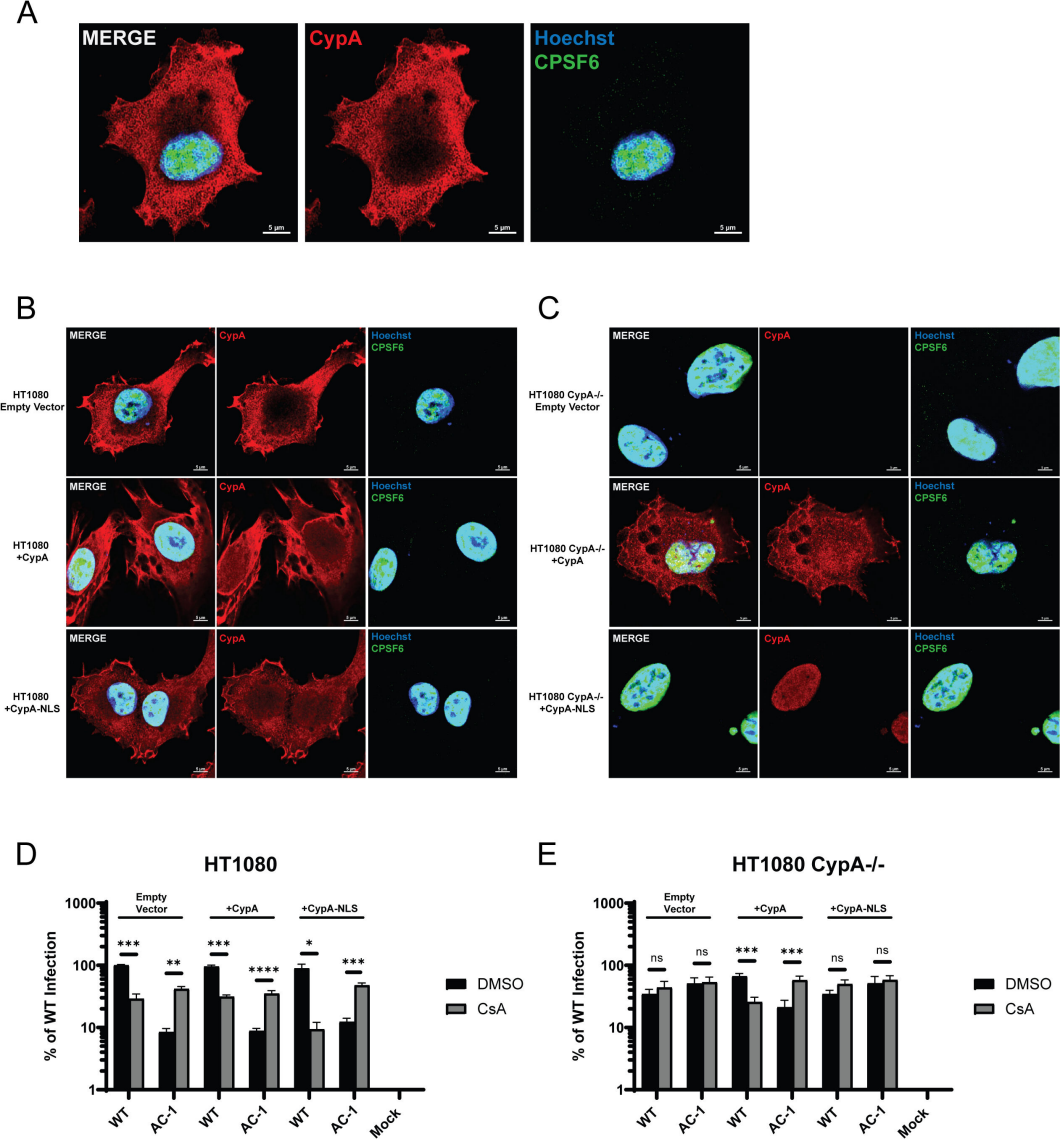
Cytoplasmic but not nuclear CypA regulates HIV-1 nuclear import and infection. (**A**) Representative confocal microscopy image of HT1080 cells stained with Hoechst (blue) and antibodies against CypA (red) and CPSF6 (green). (**B and C**) Representative images of HT1080 control cells (**B**) or HT1080 CypA*^−/^*^−^ cells (**C**) transfected with an empty control plasmid or a plasmid encoding either CypA or CypA-NLS and stained for CypA, CPSF6, and nuclei. (**D and E**) Infection of transfected HT1080 control cells (**D**) or HT1080 CypA*^−/^*^−^ cells (**E**) was measured at 48 h in media containing DMSO or 10 μM CsA (*n* = 2). Error bars represent SEM. Comparisons between infection conditions were analyzed by unpaired *t* tests. *P* values <0.05 were considered significant and significant values are denoted as *, *P* < 0.05; **, *P* < 0.01; ***, *P* < 0.001; and ****, *P* < 0.0001. ns, *P* > 0.05.

We previously showed that CypA expression in HeLa and SupT1 cells was predominantly cytoplasmic and was precluded from the nucleus and the perinuclear region overlapping with the microtubule organizing center (37). We proposed that CypA prevents premature cytoplasmic CPSF6 binding to HIV-1 capsid during trafficking toward the nucleus. Staining of CypA in HT1080 cells showed a similar expression pattern (Fig. 4A).

As increased CypA binding results in reduced HIV-1_AC-1_ nuclear import and infection, we evaluated whether cytoplasmic localization of CypA is required for nuclear import. We used a HT1080 cell line that was CRISPR-edited to delete the *PPIA* gene (64). Transfection of a plasmid encoding CypA into HT1080 control and CypA*^−/^*^−^ cells led to CypA expression throughout the cell (Fig. 4B and C). In contrast, addition of the SV40 nuclear localization signal to CypA (CypA-NLS) led to exclusive nuclear expression (Fig. 4C). Expression of empty vector, CypA, or CypA-NLS in control HT1080 cells did not affect WT HIV-1 or HIV-1_AC-1_ infection (Fig. 4D), which was consistent with other cell types (Fig. 1B, C and E). In HT1080 CypA*^−/^*^−^ cells, HIV-1_AC-1_ infection was rescued to WT HIV-1 levels regardless of CsA addition (Fig. 4E), which was similar to observations in Jurkat *PPIA^−/^*^−^ cells (Fig. 1D). While exogenous expression of CypA in HT1080 CypA*^−/^*^−^ cells reverted both WT HIV-1 and HIV-1_AC-1_ infection patterns to that of control HT1080 cells, CypA-NLS expression did not (Fig. 4E). These results suggest that CypA expression in the cytoplasm of cells regulates nuclear entry and infection.

### Increased CypA binding to HIV-1 capsid leads to reduced CPSF6 binding

**Fig 5:**
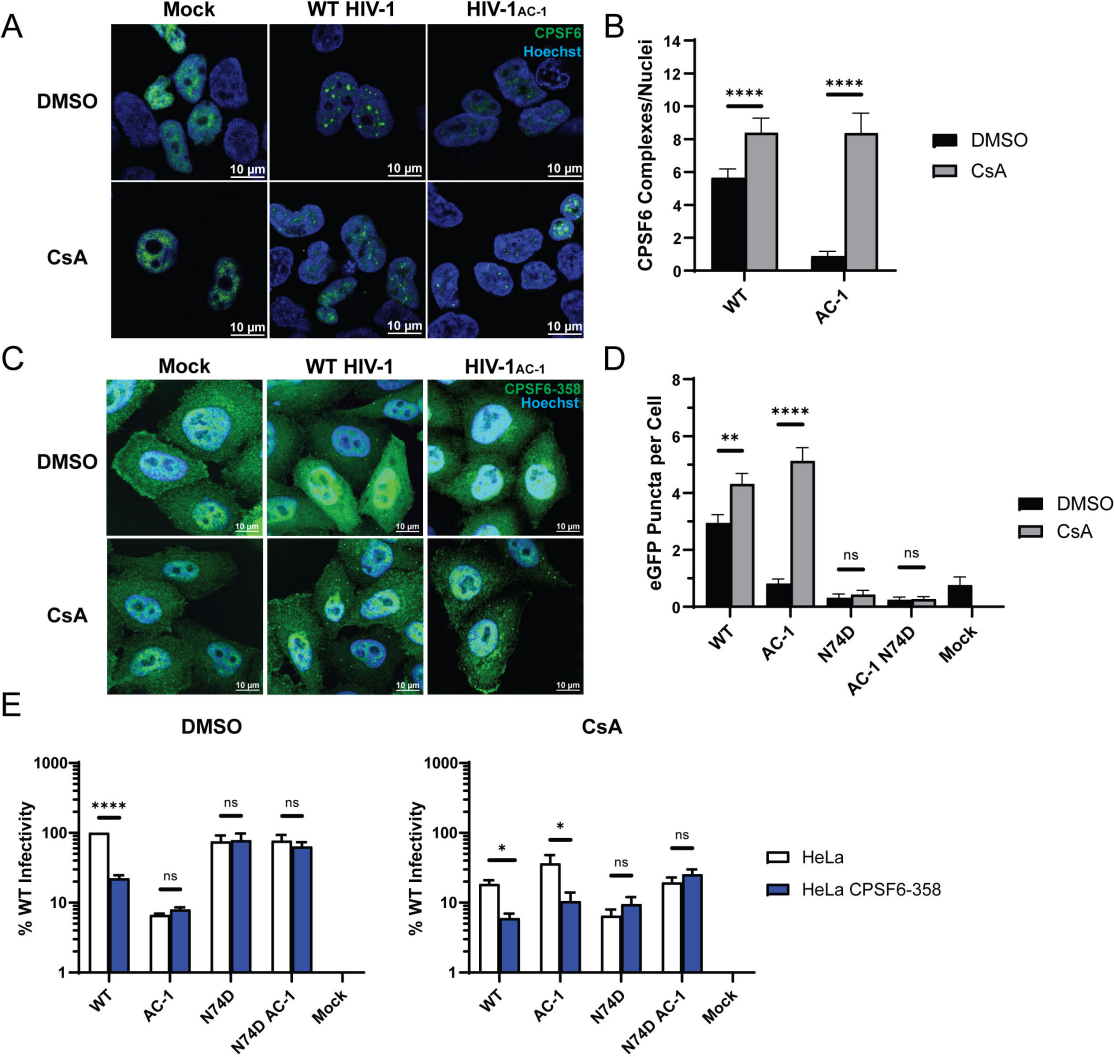
Increased CypA binding to HIV-1 capsid reduces CPSF6 binding. (**A**) Representative confocal microscopy images are shown of nuclear CPSF6-GFP higher-order complexes in HeLa cells 6 h after WT HIV-1 or HIV-1_AC-1_ infection in DMSO or 10 μM CsA. Scale bars denote 5 μm. (**B**) CPSF6-GFP higher-order complexes shown in panel A were quantified (*n* = 3). (**C**) Representative confocal microscopy images are shown of CPSF6-358-GFP higher-order complexes in HeLa cells 30 min after WT HIV-1 or HIV-1_AC-1_ infection in DMSO or 10 μM CsA. Scale bars denote 5 μm. (**D**) CPSF6-358-GFP higher-order complexes shown in panel C were quantified (*n* = 3). (**E**) HeLa cells or HeLa cells expressing CPSF6-358 were infected with WT and mutant HIV-1 in the presence of DMSO or 10 μM CsA (*n* = 3). Luciferase activity was measured after 48 h. Error bars represent SEM. Comparisons between infection conditions (**B, D, E**) were analyzed by unpaired *t* tests. *P* values <0.05 were considered significant and significant values are denoted as *, *P* < 0.05; **, *P* < 0.01; and ****, *P* < 0.0001. ns, *P* > 0.05.

Because addition of N74D to HIV-1_AC-1_ rescues infection and CypA binding limits CPSF6 binding to WT HIV-1 capsid, we investigated the interaction between HIV-1_AC-1_ capsid and CPSF6. Following WT HIV-1 nuclear entry, CPSF6 can form visible higher-order complexes in a capsid binding-dependent manner (43, 65). HeLa cells expressing GFP-CPSF6 were synchronously infected with WT HIV-1 or HIV-1_AC-1_ particles in the presence or absence of CsA for 6 h. WT HIV-1 infection resulted in visible CPSF6 higher-order complexes with or without CsA treatment, with cells averaging six or eight per nucleus, respectively (Fig. 5A and B). In contrast, few CPSF6 complexes were detected after HIV-1_AC-1_ infection unless CsA was added (Fig. 5A). Quantification of the higher-order complexes in the absence of CsA treatment showed an average of 0.9 CPSF6 complexes per nucleus (Fig. 5B), likely due to the lack of nuclear entry when CypA is bound. Addition of CsA restored formation of CPSF6 complexes by HIV-1_AC-1_ infection to an average of eight CPSF6 complexes per nucleus, comparable to WT HIV-1 levels. As a control, HeLa cells expressing GFP-CPSF6 with the F284A substitution, which prevents HIV-1 capsid binding (43, 66), were infected with WT HIV-1, resulting in 1.5 F284A CPSF6 higher-order complexes per nucleus (Fig. S5).

We next assessed whether increased CypA binding affinity prevents cytoplasmic CPSF6 binding. Truncated CPSF6-358, which lacks the RS-like domain required for TNPO3 binding, leads to cytoplasmic accumulation while maintaining HIV-1 capsid binding (40). CPSF6-358-GFP forms visible higher-order complexes in the cytoplasm upon binding to HIV-1 capsid (36), leading to aberrant uncoating, trafficking, and nuclear import, which inhibits infection (36, 37, 40). HeLa cells expressing CPSF6-358-GFP were synchronously infected with or without CsA treatment. As previously shown, WT HIV-1 produced visible CPSF6-358-GFP complexes, which increased in the presence of CsA (Fig. 5C and D). N74D HIV-1 and N74D HIV-1_AC-1_ failed to produce CPSF6-358-GFP complexes. HIV-1_AC-1_ failed to form CPSF6-358-GFP higher-order complexes in DMSO (Fig. 5C and D). However, CsA treatment restored CPSF6-358-GFP complex formation to WT HIV-1 levels, further indicating that CypA binding to HIV-1_AC-1_ capsid prevents CPSF6 binding.

To ascertain whether CPSF6-358 restricts HIV-1_AC-1_, we infected HeLa cells with or without CPSF6-358-GFP expression (Fig. 5E). WT HIV-1 infection was significantly restricted in HeLa CPSF6-358-GFP cells compared to control cells, regardless of CsA treatment. In contrast, HIV-1_AC-1_ infection was not restricted by CPSF6-358 unless CsA was present, again indicating that CPSF6-358 binding to capsid was inhibited by CypA. Both N74D HIV-1 and N74D HIV-1_AC-1_ remained insensitive to CPSF6-358, as expected given the loss of CPSF6 binding.

### Increased CypA binding to HIV-1 capsid mislocalizes integration

**Fig 6:**
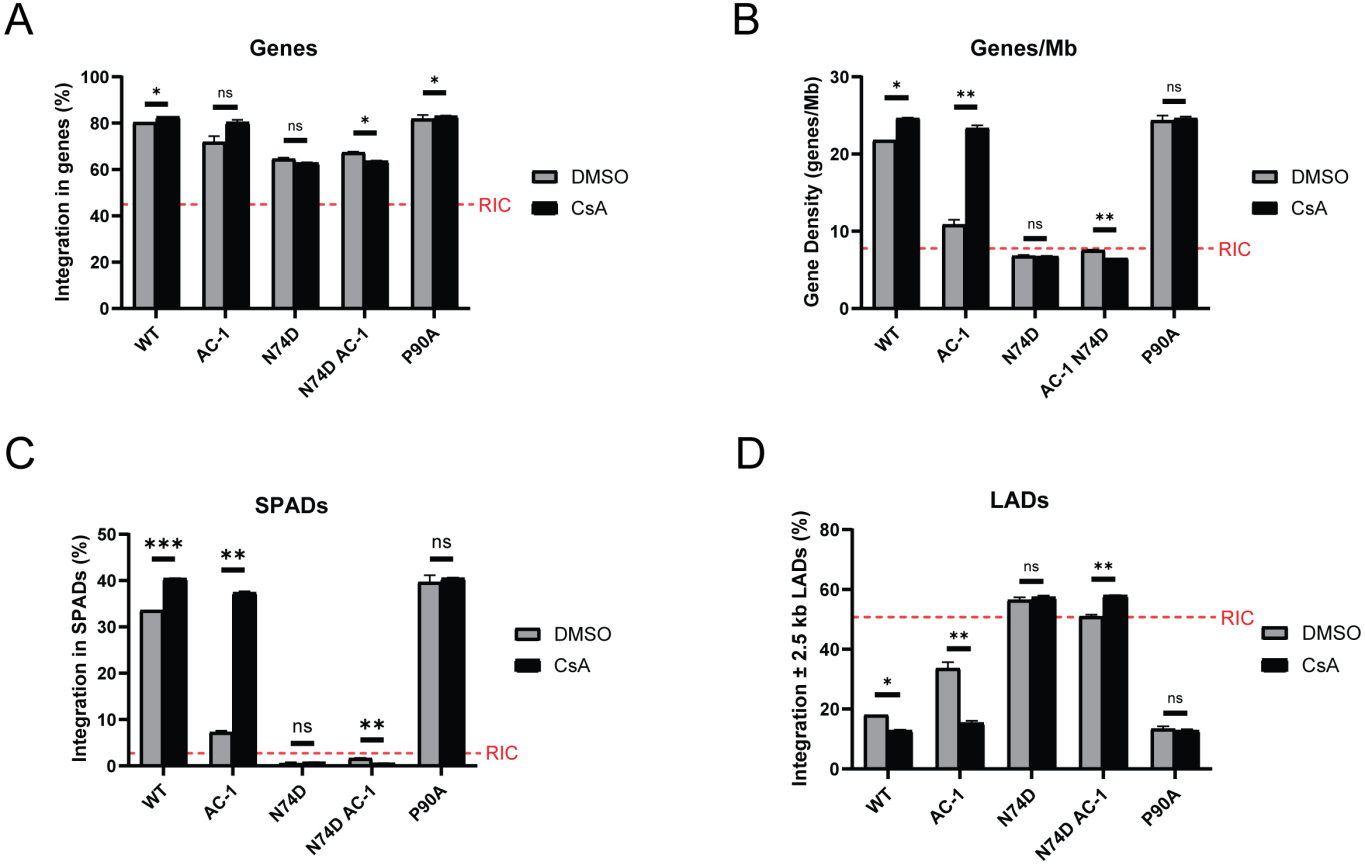
Increased CypA binding to HIV-1 capsid leads to mislocalized integration. WT and mutant HIV-1 integration sites in HeLa cells treated with DMSO or 10 μM CsA were analyzed for (**A**) genes, (**B**) gene density, (**C**) in SPADs, and (**D**) ±2.5 kb of LADs. Random integration control (RIC) values (red dashed lines) were calculated computationally via mapping 112,183 random integration sites onto human genome build 19 *in silico* following *in silico* DNA shearing (69). The infection and integration site sequencing assays were performed twice and error bars represent SEM. Comparisons between infection conditions were analyzed by unpaired *t* tests. *P* values <0.05 were considered significant and significant values are denoted as *, *P* < 0.05; **, *P* < 0.01; and ***, *P* < 0.001. ns, *P* > 0.05.

CPSF6 promotes the localization of viral cores distal from the nuclear envelope to SPADs and gene-rich chromatin, resulting in selective integration into these regions (66–70). In contrast, in the absence of CPSF6 binding, HIV-1 integration is mislocalized near the nuclear envelope to DNA containing lamina-associated domains (LADs). Because CPSF6 binding to HIV-1_AC-1_ capsid is reduced, we assessed whether HIV-1_AC-1_ integrates away from SPADs and gene-dense regions. HeLa cells were infected with HIV-1_AC-1_ or WT HIV-1 for 72 h with or without CsA. HIV-1 integration sites were amplified, sequenced, and analyzed for association with genes, gene density (genes per Mb), SPADs, and LADs, as previously described (69, 71). N74D HIV-1 and N74D HIV-1_AC-1_ were included as controls that do not bind to CPSF6, and P90A HIV-1 was included as a control that does not bind to CypA. All viruses integrated into genes above the calculated random integration control (RIC) value (Fig. 6A). WT HIV-1 and P90A HIV-1 integration sites were highly enriched in gene-dense regions (>20 genes/Mb) and within SPADs (Fig. 6B and C), while avoiding LAD-associated DNA (Fig. 6D). Notably, treatment with CsA led to significant increases in WT HIV-1 integration in genes, gene-dense regions, and SPADs, consistent with previous results (59), which may be due to increased CPSF6 binding. As expected, N74D HIV-1 and N74D HIV-1_AC-1_, which do not bind to CPSF6, disfavored gene-dense regions and SPADs, with concomitant increases in LAD-tropic targeting, regardless of CsA treatment.

Consistent with lower CPSF6 binding, HIV-1_AC-1_ infection in DMSO led to an intermediate integration pattern with an average gene density of ~10 genes/Mb and greater affinity for integration into LADs compared to WT HIV-1 (Fig. 6B and D). In contrast, CsA treatment of cells infected with HIV-1_AC-1_ rescued integration to a phenotype similar to WT HIV-1. Similar integration site patterns were seen for WT HIV-1 and HIV-1_AC-1_ after infection of HT1080 CypA*^−/^*^−^ cells, with and without CypA expression (Fig. S6). While CypA expression in CypA*^−/^*^−^ cells resulted in HIV-1_AC-1_ integration patterns that were more similar to WT HIV-1, CypA-NLS did not. Collectively, these results suggest the importance of capsid binding to both cytoplasmic CypA and CPSF6 for optimal integration.

## DISCUSSION

Previously, the HIV-1_AC-1_ mutant was produced to increase CypA binding affinity to capsid to study immune sensing of HIV-1 infection in myeloid cells (44). Lahaye et al. determined that this mutant displayed approximately 1.5-fold increased CypA binding, which significantly inhibited HIV-1 infection at the nuclear import step, as measured by 2-LTR circles (45). However, infection of OMK cells demonstrates a greater increase in CypA binding by HIV-1_AC-1_. By measuring 2-LTR circles with the NIK assay, we also showed that nuclear translocation of HIV-1_AC-1_ capsid is significantly reduced. The infectivity defect was abrogated by treatment of cells with an inhibitor of CypA binding to CA, knockout of CypA in cells, or addition of a CA amino acid substitution to prevent CypA binding but not by deletion of the NUP358 CHD. Collectively, this demonstrates that cytoplasmic CypA binding to capsid directly mediates nuclear import and promotes replication.

**Fig 7:**
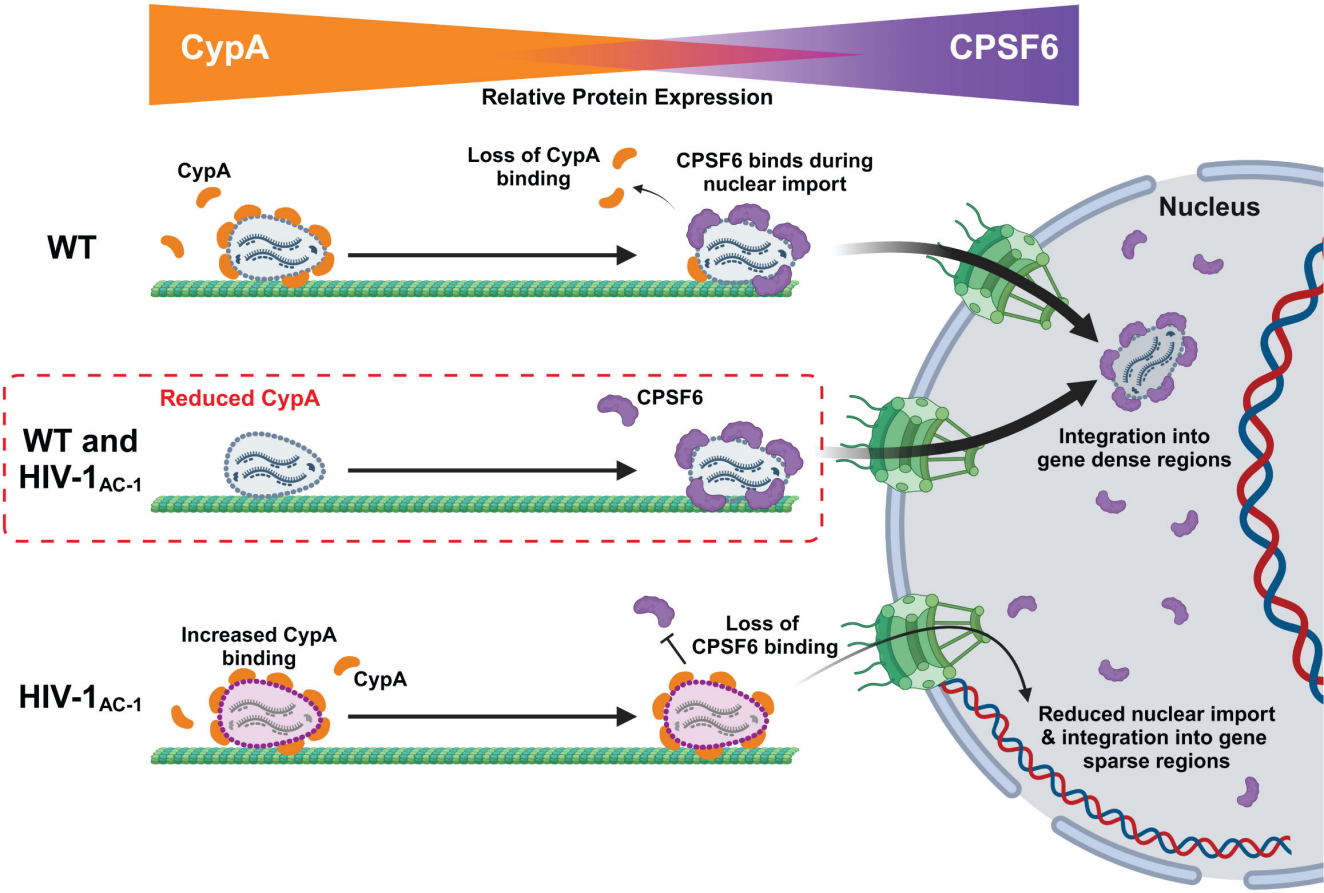
Schematic model of HIV-1_AC-1_ replication and restriction. Cytoplasmic WT HIV-1 engages with CypA to sterically block premature CPSF6 binding. As the HIV-1 capsid approaches the nucleus, CypA concentration decreases, leading to an exchange of CypA for CPSF6 binding in preparation for nuclear import. HIV-1_AC-1_ displays increased CypA binding that results in failed nuclear import due to inability to engage cytoplasmic CPSF6.

Introduction of a mutation that prevents CPSF6 binding to capsid, N74D, rescued HIV-1_AC-1_ infectivity, suggesting that the phenotype is caused by capsid binding to CPSF6. Our previous studies showed that CPSF6 binding to HIV-1 capsid mediated cytoplasmic trafficking and uncoating (36, 37). Additionally, HIV-1 nuclear import is enhanced by CPSF6 binding to TNPO3 for nuclear localization (40, 42, 67, 72). Furthermore, CypA binding to HIV-1 capsid shielded it from binding to cytoplasmic CPSF6 (36, 37) and TRIM5α (31, 32). While localization of CPSF6 was predominantly nuclear with some higher-order complexes detected in the cytoplasm near the nucleus and associated with microtubules, CypA localized to the cell periphery and was excluded from regions of CPSF6 expression. With increased CypA binding to HIV-1_AC-1_ capsid, the interaction with CPSF6 is compromised in the cytoplasm and the nucleus. Furthermore, relocalization of CypA exclusively to the nucleus led to infection phenotypes of WT HIV-1 and HIV-1_AC-1_ similar to those in cells lacking CypA. Thus, our model of optimal HIV-1 replication suggests that CypA binds to the incoming capsid at the periphery of the cell and the availability of CypA decreases near the nucleus, allowing CPSF6 to engage with the capsid, leading to a competitive exchange of capsid binding host factors that is spatiotemporally regulated (Fig. 7).

At the nucleus, HIV-1 capsid acts like a nuclear transport receptor, binding to phenylalanine-glycine (FG) repeats of nucleoporin condensates to enter the nucleus via NPCs (8, 9). CPSF6 binding to CLPs competes with nucleoporin FG binding (8), likely in a similar manner of sequential host factor binding to HIV-1 capsid. Previous studies proposed that, while CPSF6 is not required for entry through the NPC, it is needed for capsid uncoating and migration away from the inner NPC within the nucleus (67, 73, 74). CypA binding to capsid is required for interaction with inner NPC proteins POM121 and Nup35 (65), further suggesting a direct role of CypA in nuclear entry. One can envision a precise sequence of host factor binding to HIV-1 capsid to move it from the periphery of the cell to the cytoplasmic face of the NPC and then through the NPC. However, in the case of HIV-1_AC-1_, nuclear import through the NPC may be further hindered due to excessive CypA binding. Investigation of this mutant with different NUP-FG condensates with and without CypA would provide additional information on the role of CypA in shielding NPC components.

After entry into the nucleus, HIV-1 capsid interacts with the CPSF6 FG dipeptide/prion-like domain (41, 75, 76) to enable viral replication complexes to be trafficked further into the nucleus for integration into gene-rich SPADs (43, 66, 67). Surprisingly, HIV-1_AC-1_ had similar *in vitro* capsid stability as WT HIV-1. This was validated in cells by the introduction of CsA at different time points, which led to rescue of HIV-1_AC-1_ infectivity up to 6 h post-infection, indicating that the HIV-1_AC-1_ capsid is likely largely intact. In addition, HIV-1_AC-1_ completed reverse transcription, which has been shown to occur even in hyperstable capsids during infection (75). Beyond 6 h, HIV-1_AC-1_ infectivity could not be rescued, suggesting that an irreversible change occurred in the capsid that could not be overcome even upon the loss of CypA binding. Any HIV-1_AC-1_ capsids that entered the nucleus, perhaps during mitosis, appeared not to engage nuclear CPSF6 in a manner consistent with WT HIV-1, leading to misintegration near the nuclear membrane in LADs. This is similar to N74D HIV-1 that is unable to bind to CPSF6 (66, 69). Interestingly, the mislocalization of HIV-1_AC-1_ integration was not as pronounced as N74D HIV-1, suggesting that some CPSF6 binding can occur in the nucleus. In contrast, with a loss of cytoplasmic CypA binding, HIV-1_AC-1_ integrated preferentially into gene-dense regions and SPADs while avoiding gene-sparse LADs, similar to WT HIV-1.

Our factor exchange model focused on the spatial and temporal aspects of host factors binding to HIV-1 capsid, which are influenced by mutations. Surprisingly, the introduction of the five CA substitutions to create HIV-1_AC-1_ results in a relatively small change in binding affinity to CypA (44) that significantly impacts viral replication. This suggests that a weaker binding affinity of CypA to HIV-1 capsid allows a proper host factor exchange with CPSF6 for optimal nuclear import and integration. Analysis of 20,000 HIV-1 group M *gag* sequences showed that five of the six HIV-1_AC-1_ substitutions are highly polymorphic (77). Proline is the consensus residue at position 92 and Ile86, Leu91, and Leu96, all HIV-1_AC-1_ changes, are observed at up to 10% prevalence. It is possible that CypA binding to HIV-1 capsid may be variable in clinical isolates, which may lead to differential host factor binding during replication and could affect pathogenesis. Our model incorporates spatiotemporal regulation of host protein composition on capsids that promotes or restricts HIV-1 replication. Importantly, this model can be expanded beyond CypA and CPSF6 to other cell factors that interact with capsid during infection.

## MATERIALS AND METHODS

### Cell lines and primary cultures

HeLa, HEK293T, HT1080, HT1080 NUP358delCyp (63), and HT1080 CypA*^−/^*^−^ cells (64) were grown in culture with Dulbecco's modified Eagle medium (DMEM; Thermo Fisher) supplemented with 10% fetal bovine serum (FBS; Atlanta Biologicals) and 100 mg/mL penicillin, 100 mg/mL streptomycin, and 2 mM l-glutamine (PSG; Thermo Fisher) at 37°C and 5% CO_2_. Jurkat cells and Jurkat *PPIA^−/^*^−^ cells (obtained through the NIH HIV Reagent Program; contributed by Drs. Braaten and Luban) were cultured in RPMI medium supplemented with 10% FBS and PSG. Primary CD4+ T cells were isolated from whole blood (BioIVT) using the CD4+ T Cell Isolation Kit (Miltenyi Biotec) and were cultured in RPMI medium containing 10% FBS, PSG, and 20 IU/mL IL-2 and stimulated with 2 mg/mL PHA for 48 h prior to infection. GHOST-R3/X4/R5 cells (78) were maintained in DMEM medium containing 10% FBS, PSG, 1 mg/mL puromycin, 100 mg/mL hygromycin, and 500 mg/mL geneticin.

### Plasmids and viruses

Viruses were produced by transfection of HEK293T cells using either Lipofectamine 2000 (Invitrogen) or polyethylenimine (Polysciences). The plasmids encoding replication-defective HIV-1 with firefly luciferase or mNeonGreen in place of *nef* (pNLdE-luc and pNLdE-mNeon, respectively) and pL-VSV-G were used to produce pseudotyped HIV-1 (40). The HIV-1_AC-1_ mutations were introduced into WT and N74D pNLdE-luc using the Gibson Assembly Cloning kit (New England Biolabs) and primers shown in Table S1. The P90A substitution was generated via the QuikChange Mutagenesis Kit (Agilent) using primers shown in Table S1. Fluorescently labeled IN was packaged into viruses using the pVpr-mRuby3-IN or pVpr-mNeon-IN plasmids during transfection (36). Labelling of viruses was confirmed by imaging viruses in MatTek dishes by total internal reflection fluorescence (TIRF) microscopy. Lentiviral vectors encoding control or TRIM5α miRNAs (pAPM-D4-miR30-L1221 or pAPM-D4-miR30-TRIM5α; Addgene) were produced by co-transfection with the packaging plasmid pcHelp (79) and pCMV-VSV-G (31). HIV-1 titers were determined in GHOST-R3/X4/R5 cells, and total virus was quantified by p24 ELISA (XpressBio). N-MLV was produced by transfection of the following plasmids: pCIG3-N (a gift from Jonathan Stoye) (80), pFB-Luc vector (Agilent), and pCMV-VSV-G. All viruses were harvested by passing cell supernatants through a 0.45 μm filter.

HIV-1 CA coding region for WT HIV-1 was cloned into the pET11a vector for *in vitro* assays. The HIV-1_AC-1_ mutations were generated using the QuikChange Lightning kit (Agilent) with primers shown in Table S1.

The plasmid pLVX-hNUP62Fv2GFP for the NIK assay was a gift from Edward Campbell (17). Plasmids pLBCX-GS-HA-eGFP-CPSF6 and pLBCX-GS-HA-eGFP-CPSF6/F284A to express GFP fusions of CPSF6 (WT or F284A) were constructed in plasmid pLB(N)CX, as previously described (66). CypA was PCR-amplified from cDNA produced with random hexamers from HeLa cell RNA. The PCR product was Topo cloned into the plasmid pcDNA3.1 (Thermo Fisher) to produce pCypA. To add the SV40 NLS to the C-terminus of CypA, we annealed partially complimentary primers encoding the SV40 NLS (Table S1) at 95°C for 4 min and 70°C for 10 min, followed by cooling them to room temperature over 4 h. The annealed primers formed overhangs consistent with BamHI and NotI restriction digestion sites and were gel-purified and cloned into pCypA-DsRed (81) to replace DsRed with the NLS, producing pCypA-NLS.

### CA protein purification and assembly

WT HIV-1 and HIV-1_AC-1_ CA proteins were expressed and purified as previously described (82). Conical CLPs were assembled by incubating 500 μM CA in 4  mM IP6 and 100  mM MES (pH 6) at 37°C for 1  h (83). Tubular CLPs were assembled by incubating 350 μM CA in 5  mM IP6, 20  mM Tris (pH 8), and 100 mM NaCl, 37°C for 1 h (83).

### Co-pelleting assays and immunoblotting

CypA was purified as previously described (84). WT and HIV-1_AC-1_ tubes and CLPs (325 µM in terms of CA subunits, equivalent to 54.1 µM in terms of CA hexamers) were incubated without or with 23 µM CypA, for 1 h at room temperature. Different ratios of CA:CypA were assayed by incubating 20 µM CA subunits with 2, 20, or 200 µM CypA overnight at 4°C. After incubation, input samples were collected. Pellet and supernatant fractions were separated by centrifugation at 21,000 × g, for 10 min, at 4°C. Fractions were analyzed by SDS-PAGE, using Mini-Protean 4–20% gradient gels (Biorad), and Coomassie staining.

Proteins separated by SDS-PAGE were transferred to a PVDF membrane (100 V, 1 h). Membranes were blocked with 5% milk in PBS-Tween (PBS with 0.05% Tween 20) for 1 h. Primary antibody rabbit polyclonal anti-CypA (GeneTex, C1C3, 1:5,000) was incubated with membranes for 1 h. Membranes were washed three times with PBS-Tween. Secondary antibody IRDye-680RD goat anti-rabbit IgG (LI-COR, 1:20,000) was incubated with membranes for 1 h. All incubations were performed at room temperature. After washes, membranes were exposed to the IR700 detection channel, using a c600 imager system (Azure Biosystems).

### HIV-1 infectivity assays

HeLa cells (5 × 10^4^) were infected in technical duplicates with VSV-G pseudotyped HIV-1 (10 ng p24) in the presence of 5–10 μM CsA in DMSO or equivalent concentrations of DMSO. For growth arrest, cells were cultured for 18 h with 1 μg/mL aphidicolin. For knockdown of TRIM5α, cells were transduced with lentiviral vectors expressing control or TRIM5α miRNA for 48 h and selected with 2 μg/mL puromycin prior to infection with HIV-1 or N-MLV in the presence or absence of CsA. Cells were either lysed or fixed with 2% paraformaldehyde (PFA) 48 h later. Infectivity was measured by either luciferase or mNeon expression. HT1080 and HT1080 CypA*^−/^*^−^ cells were transfected with pcDNA3.1, pCypA, or pCypA-NLS 48 h prior to HIV-1 infection in the presence or absence of CsA.

### CA retention assay

The CA retention assay was performed on WT HIV-1 and CA mutants as previously described (52). Briefly, chamber slides (Greiner) were treated with Cell-Tak (Corning). HIV-1 (5 ng p24) containing mRuby3-IN was added to each well in 100 mM NaCl, 10 mM Tris-HCl, and 1 mM EDTA (pH 7.4). Samples were mounted in gelvatol and imaged by TIRF on a Nikon Eclipse Ti microscope. Six fields per condition were imaged and analyzed with Nikon Elements software.

### Imaging of CPSF6 and CypA

HeLa cells expressing CPSF6-358-eGFP or eGFP-CPSF6 were synchronously infected with HIV-1 in the presence or absence of CsA and imaged as previously described (36). Cells were fixed in 2% PFA. Fixed samples were permeabilized with 0.1% Triton-X100 before staining with Hoechst. Imaging was performed on a Nikon A1 confocal microscope with a motorized piezo Z stage. Z-stacks were created before enumerating nuclei and CPSF6 higher-order complexes in 12 fields of view using Elements.

Transfected HT1080 and HT1080 CypA*^−/^*^−^ cells were fixed with 2% PFA and permeabilized with 0.1% Triton-X100 before staining with mouse anti-CypA (Abcam) and rabbit anti-CPSF6 (Novus) antibodies. Some samples were stained with AlexaFluor488-phalloidin (Thermo Fisher). Samples were then stained with either donkey anti-mouse-Cy3 (Jackson Immuno Research) or donkey anti-rabbit-AlexaFluor488 (Thermo Fisher), respectively, and Hoechst before mounting samples and imaging on a Nikon A1 confocal microscope.

### NIK assay

HeLa cells expressing hNUP62Fv2GFP were growth arrested with 1 μg/mL aphidicolin for 24 h prior to synchronous infection with equal amounts of p24 with or without of CsA. At each timepoint, media containing 1.5 μM AP20187 (MedChemExpress) was added to the cells. Media was replaced at 24 h, and cells were lysed 24 h later for measurement of luciferase. Infectivity of each time point was normalized to cells infected in the absence of AP20187.

### Measurement of HIV-1 reverse transcripts and 2-LTR circles

HeLa cells treated with or without CsA were infected with DNase I-treated HIV-1 (10 ng p24). Rilpivirine (1 μM; NIH HIV Reagent Program) was added as a control to parallel cultures to prevent reverse transcription and control for plasmid carryover from transfection to produce virus. After 24 h, the cells were removed with trypsin-EDTA, washed, and pelleted. Genomic DNA was extracted using the QIAmp DNA Mini Kit (Qiagen). Reverse transcript products and 2-LTR circles were measured by quantitative PCR (qPCR) using primers and probes (Table S2), as previously described (85).

### Integration site analysis

HeLa cells treated with or without CsA were infected with DNase-treated HIV-1 at a multiplicity of infection of 1 and were trypsinized 3 days post-infection. Genomic DNA was extracted from all samples using the QIAmp DNA Mini Kit. Integration libraries were prepared using ligation-mediated PCR (LM-PCR) essentially as described previously (71, 86). In brief, DNA (5 µg) was digested and ligated with linkers using NEBNext Ultra II FS DNA Library Prep Kit following the manufacturer's protocol and as previously described (87). Ligation was performed in three separate batches for 16 h at 20°C. Purified ligation products were subjected to semi-nested PCR using linker and LTR-specific primers to amplify viral-host integration junctions. Purified PCR products were subjected to 150 bp paired-end Illumina sequencing at Genewiz.

HT1080 cells were infected with a U3-tagged HIV-1 construct to discriminate the infecting virus from the lentiviral vector used during cell line construction (66, 88 ). HT1080 cell DNA was fragmented with AvrII, NheI, SpeI, and BamHI restriction endonucleases, after which four independent ligation reactions were processed as described above. Virus-host junctions were amplified using semi-nested PCR as described above. Purified PCR products were subjected to 150 bp paired-end sequencing using a NextSeq 2000 Illumina sequencer.

Illumina raw reads were processed, and integration sites were determined against the hg19 human genome and analyzed with various genomic features, as previously described (69, 88). Random integration control (RIC) data sets were generated *in silico* using methods to mimic respective wet-bench manipulations of genomic DNA (69, 88).

### Statistical analysis

Statistical analysis for all was performed in Prism (GraphPad) with statistical tests described in the figure legends. Experiments were completed with technical replicates and two to three experimental replicates.

## Data Availability

Raw sequences are available at the National Center for Biotechnology Information (accession no. PRJNA1098037).

## SUPPLEMENTAL MATERIAL

Supplemental material mbio.00169-25-s0001.pdf Tables S1 and S2 and Fig. S1 to S6.

ASM does not own the copyrights to Supplemental Material that may be linked to, or accessed through, an article. The authors have granted ASM a non-exclusive, world-wide license to publish the Supplemental Material files. Please contact the corresponding author directly for reuse.
