# Supplementary material for: Spatiotemporal binding of cyclophilin A and CPSF6 to capsid regulates HIV-1 nuclear entry and integration
Source: mBio. 2025 Feb 27;16(4):e00169-25. doi: 10.1128/mbio.00169-25 (PMC11980554; doi:10.1128/mbio.00169-25)
Supplement: Supplemental material — Tables S1 and S2 and Fig. S1 to S6. [file mbio.00169-25-s0001.pdf]

Table S1: Primers used for cloning and mutagenesis of HIV-1 and CypA constructs

Name	Sequence (5' to 3')	Use
F-HIV-1_{AC-1}	CATCCAATACATGCAGGGCCTCTTCCAGCAGGCCAGT TGAGAGAACCAAG	Site Directed Mutagenesis for AC-1 pET11a
R-HIV-1_{AC-1}	CTTGGTTCTCTCAACTGGCCTGCTGGAAGAGGCCCTG CATGTATTGGATG	
NL43-CAac-1 F	GGATAGATTGCATCCAATACATGCAGGGCCTCTT CCAGCAGGCCAGTTGAGAGAACCAAGGGG	Gibson Assembly for AC-1 pNLdE-Luc
NL43-CAac-1 R	CCCCTTGGTTCTCTCAACTGGCCTGCTGGAAGA GGCCCTGCATGTATTGGATGCAATCTATCC	
KanR-NcoI F	CCCCTTGGTTCTCTCAACTGGCCTGCT GGAAGAGGCCCTGCATGTATTGGATGCAATCTATCC	Gibson Assembly for AC-1 pNLdE-Luc
KanR-NcoI R	CCACCATGATATTCGGCAAGCAGGCAT CGCCATGGGTCACGACGAGATCCTCGCCGTCG	
P90A-ac-1 F	CCAATACATGCAGGGGCTCTTCCAGCAGGCCAG	Site Directed Mutagenesis for P90A AC-1 NLdE-Luc
P90A-ac-1 R	CTGGCCTGCTGGAAGAGCCCCTGCATGTATTGG	
CypA F	AACCGTGTACTATTAGCCATGG	PCR amplification of CypA from human cDNA
CypA R	AGTCAAACTTATTCGAGTTGTCC	
BamHI-NLS F	GGATCCACCGGTCGCCACCCCGAAAAAAAAACGCAA AGTGGAAGATCCGTAGC	Cloning of NLS onto CypA
NLS-NotI R	GCGGCCGCTACGGATCTTCCACTTTGCGTTTTTTTTTC GGGGTGGCGACCGGTG	

Table S2: Primers for quantitative PCR

Name	Sequence (5' to 3')
gag F	GCCTGGGAGCTCTCTGGCTAA
gag R	GCCTTGTGTGTGGTAGATCCA
gag Probe	FAM-AAGTAGTGTGTGCCCGTCTGTTGTGTGACTC-TAMRA
2-LTR F	TTCGCAGTTAATCCTGGCCTT
2-LTR R	GCACACAATAGAGGACTGCTATTGTA
2-LTR Probe	FAM-TAGAGACATCAGAAGGCTGTAGACAAA-TAMRA

Supplemental Figure Legends

Figure S1. (A) CA sequences (amino acids 70-100) are compared between WT HIV-1 and mutants. WT CA and HIV-1_{AC-1} IP6-assembled **(**B,D**)** CLPs and **(**C,E**)** tubes were incubated without or with purified CypA (14:1 ratio) at room temperature for 1 h. **(**B,C**)** I – input, P – pellet, and S - supernatant fractions were analyzed using Coomassie stained SDS-PAGE gels. **(**D,E**)** Immunoblotting for CypA indicates similar amounts of co-precipitated CypA in the presence of WT CA or HIV-1_{AC-1} CA tubes and CLPs.

Figure S2. (A) Infectivity of WT and mutant HIV-1 (10 ng p24) was determined after 48 h by luciferase activity in DMSO or 10 μM CsA in HeLa cells (n = 3). (B) N-MLV infection was determined after 48 h by luciferase activity in HeLa cells transduced with lentiviruses expressing control or TRIM5α miRNA (n = 2). Error bars represent SEM. Comparisons between infection conditions were analyzed by unpaired t tests. (C, D) DMSO or 10 μM CsA was added at different time points to HeLa cells synchronously infected with WT HIV-1 (C) or HIV-1_{AC-1} (D). The assay was performed three times. Error bars represent SEM and unpaired t-tests were performed for comparisons between conditions at each time point. P values < 0.05 were considered significant and significant values are denoted as *, $p < 0.05$ and **, $p < 0.01$. ns, $p > 0.05$.

Figure S3. Nuclear import kinetics were determined for (A) WT HIV-1 and (B) HIV-1_{AC-1} infection of HeLa cells in DMSO or 10 μM CsA containing media at different time points in three independent experiments. Infectivity was determined by luciferase activity (relative luciferase units, RLU). Error bars represent SEM.

Figure S4. Infectivity of WT and mutant HIV-1 (10 ng p24) was determined after 48 h by luciferase activity in DMSO or 5-10 μM CsA in HT1080 cells and HT1080 NUP358delCyp cells in two independent experiments. Error bars represent SEM. ns, $p > 0.05$.

Figure S5. (A) Representative confocal microscopy images are shown of nuclear WT or F284A CPSF6-GFP higher order complexes in HeLa cells 6 h after WT HIV-1 infection. Scale bars denote 5 μm. (B) WT and F284A CPSF6-GFP higher order complexes shown in A were quantified ($n = 3$). Error bars represent SEM. The comparison of WT and F284A CPSF6-GFP puncta was analyzed by an unpaired t test. P values of < 0.05 were considered significant and significant values are denoted as ****, $p < 0.0001$.

Figure S6. (A) Representative images of HT1080 CypA$^{-/-}$ cells transfected with an empty control plasmid or a plasmid encoding either CypA or CypA-NLS and stained for CypA (red), actin filaments (phalloidin; green), and DNA (Hoechst; blue). (B-F) Cells shown in A were infected with WT HIV-1, HIV-1$_{AC-1}$, and P90A HIV-1$_{AC-1}$ in duplicate for 48 h. (B) Infectivity, as measured by luciferase activity, is shown for each condition. HIV-1 integration sites were analyzed for (C) number of mapped sites, (D) in genes, (E) average gene density, and (F) in SPADs. Random integration control (RIC) values (red dashed lines) were calculated computationally via mapping integration sites onto human genome build 19 *in silico* following *in silico* DNA shearing (88). Error bars represent SEM. Comparisons between infection conditions were analyzed by unpaired

t tests. P values < 0.05 were considered significant and significant values are denoted as *, $p < 0.05$. ns, $p > 0.05$.

Figure S1

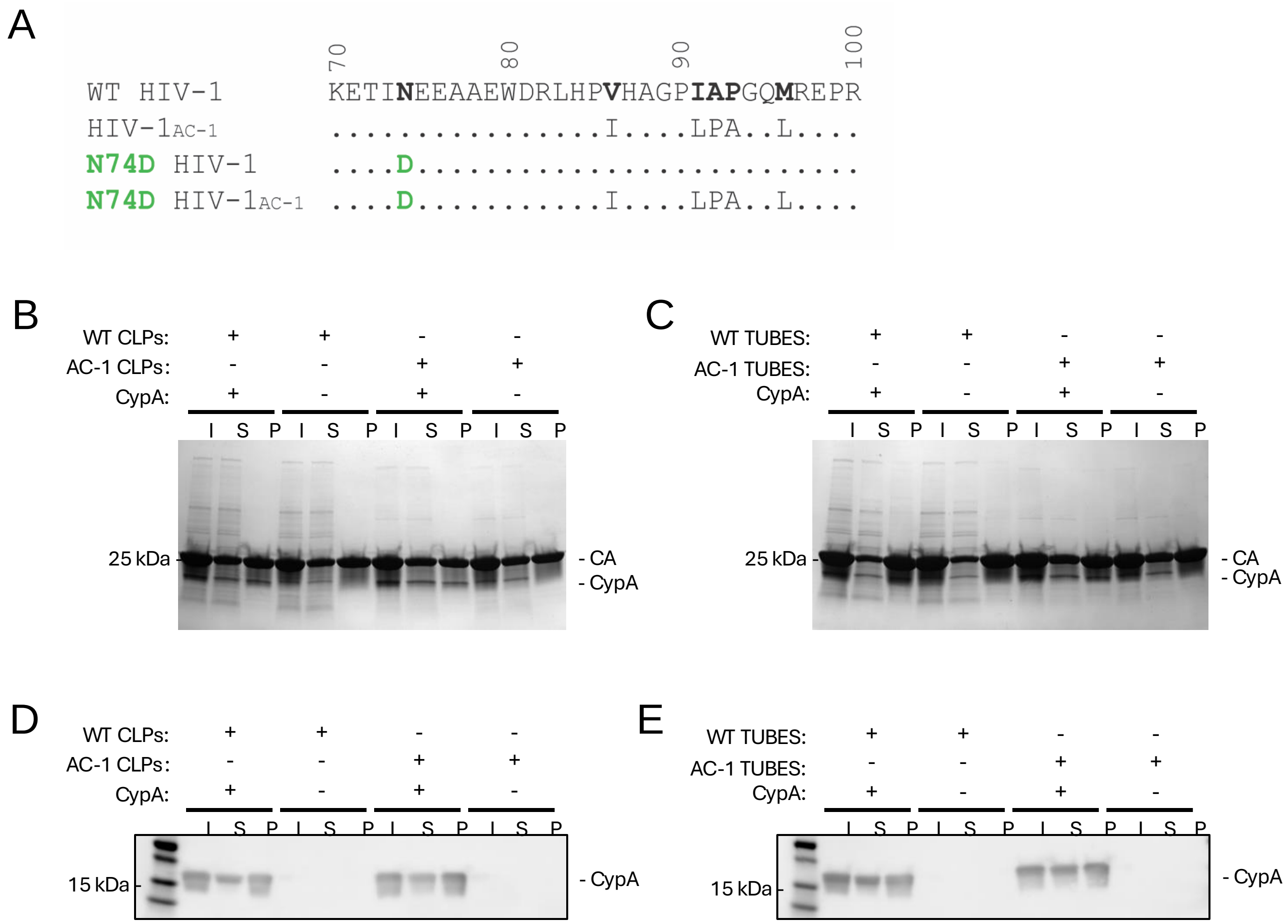
A
70
80
90
100
WT HIV-1 KETINEEAAEWDRLHPVHAGPIAPGQMREPR
HIV-1AC-1 ................I....LPA..L....
N74D HIV-1 ....D..........................
N74D HIV-1AC-1 ....D...........I....LPA..L....
B
WT CLPs: + + - -
AC-1 CLPs: - - + +
CypA: + - + -
I S P I S P I S P I S P
25 kDa
- CA
- CypA
C
WT TUBES: + + - -
AC-1 TUBES: - - + +
CypA: + - + -
I S P I S P I S P I S P
25 kDa
- CA
- CypA
D
WT CLPs: + + - -
AC-1 CLPs: - - + +
CypA: + - + -
I S P I S P I S P I S P
15 kDa
- CypA
E
WT TUBES: + + - -
AC-1 TUBES: - - + +
CypA: + - + -
I S P I S P I S P I S P
15 kDa
- CypA

Figure S2

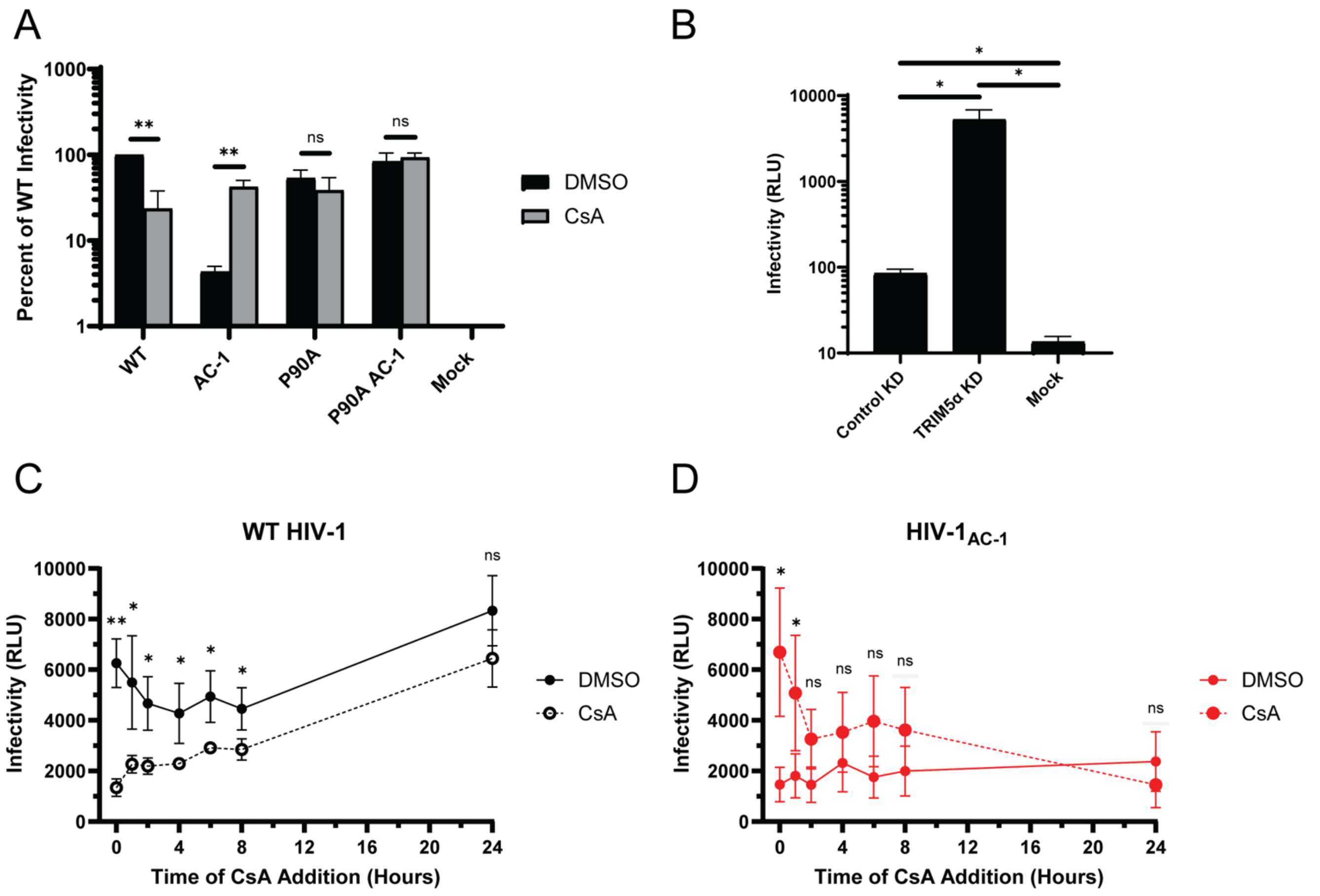

A
Percent of WT Infectivity
1000
100
10
1
**
**
ns
ns
WT
AC-1
P90A
P90A AC-1
Mock
DMSO
CsA
B
Infectivity (RLU)
10000
1000
100
10
*
*
*
Control KD
TRIM5α KD
Mock
C
WT HIV-1
Infectivity (RLU)
10000
8000
6000
4000
2000
0
**
*
*
*
*
*
ns
0
4
8
12
16
20
24
Time of CsA Addition (Hours)
DMSO
CsA
D
HIV-1AC-1
Infectivity (RLU)
10000
8000
6000
4000
2000
0
*
*
ns
ns
ns
ns
ns
0
4
8
12
16
20
24
Time of CsA Addition (Hours)
DMSO
CsA

Figure S3

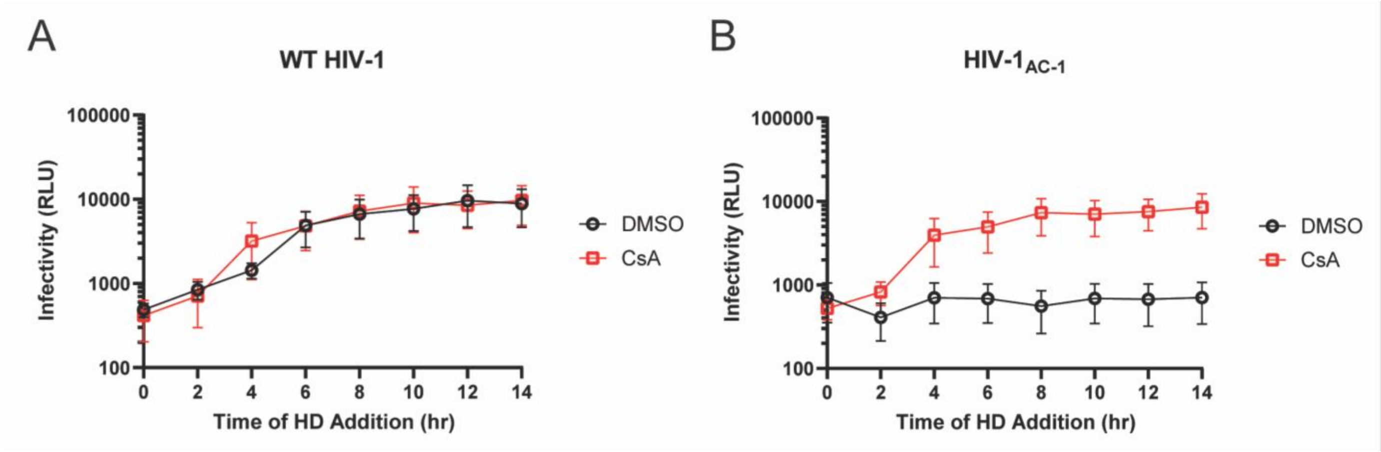
A
WT HIV-1
100000
10000
1000
100
Infectivity (RLU)
0
2
4
6
8
10
12
14
Time of HD Addition (hr)
DMSO
CsA
B
HIV-1AC-1
100000
10000
1000
100
Infectivity (RLU)
0
2
4
6
8
10
12
14
Time of HD Addition (hr)
DMSO
CsA

Figure S4

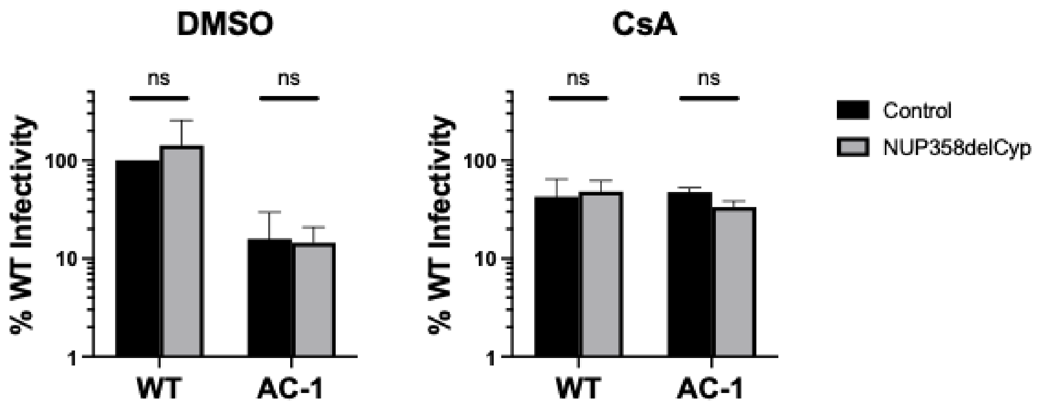
DMSO
CsA
ns
ns
ns
ns
% WT Infectivity
% WT Infectivity
100
10
1
100
10
1
WT
AC-1
WT
AC-1
Control
NUP358delCyp

Figure S5

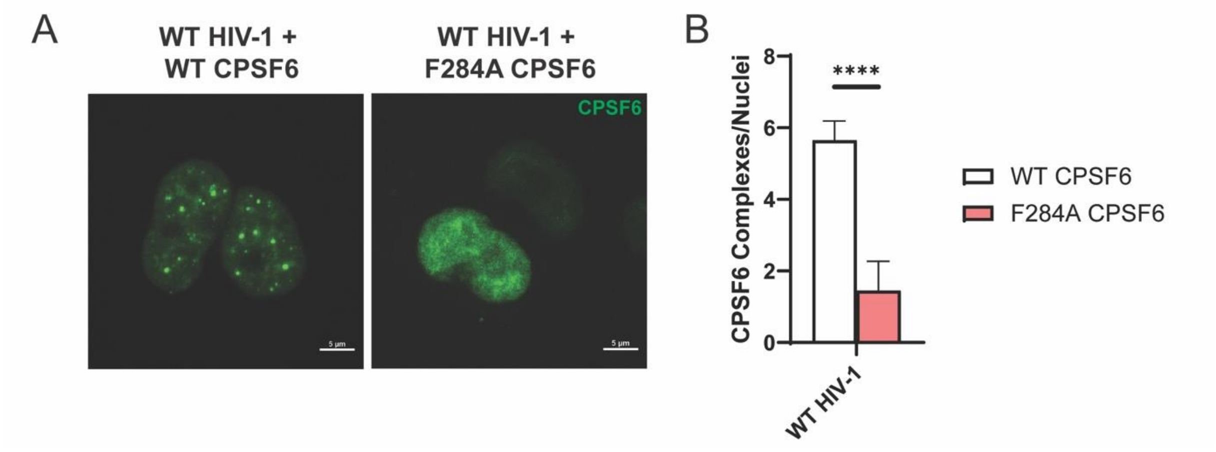
A
WT HIV-1 +
WT CPSF6
WT HIV-1 +
F284A CPSF6
CPSF6
B
CPSF6 Complexes/Nuclei
8
6
4
2
0
****
WT HIV-1
WT CPSF6
F284A CPSF6

Figure S6

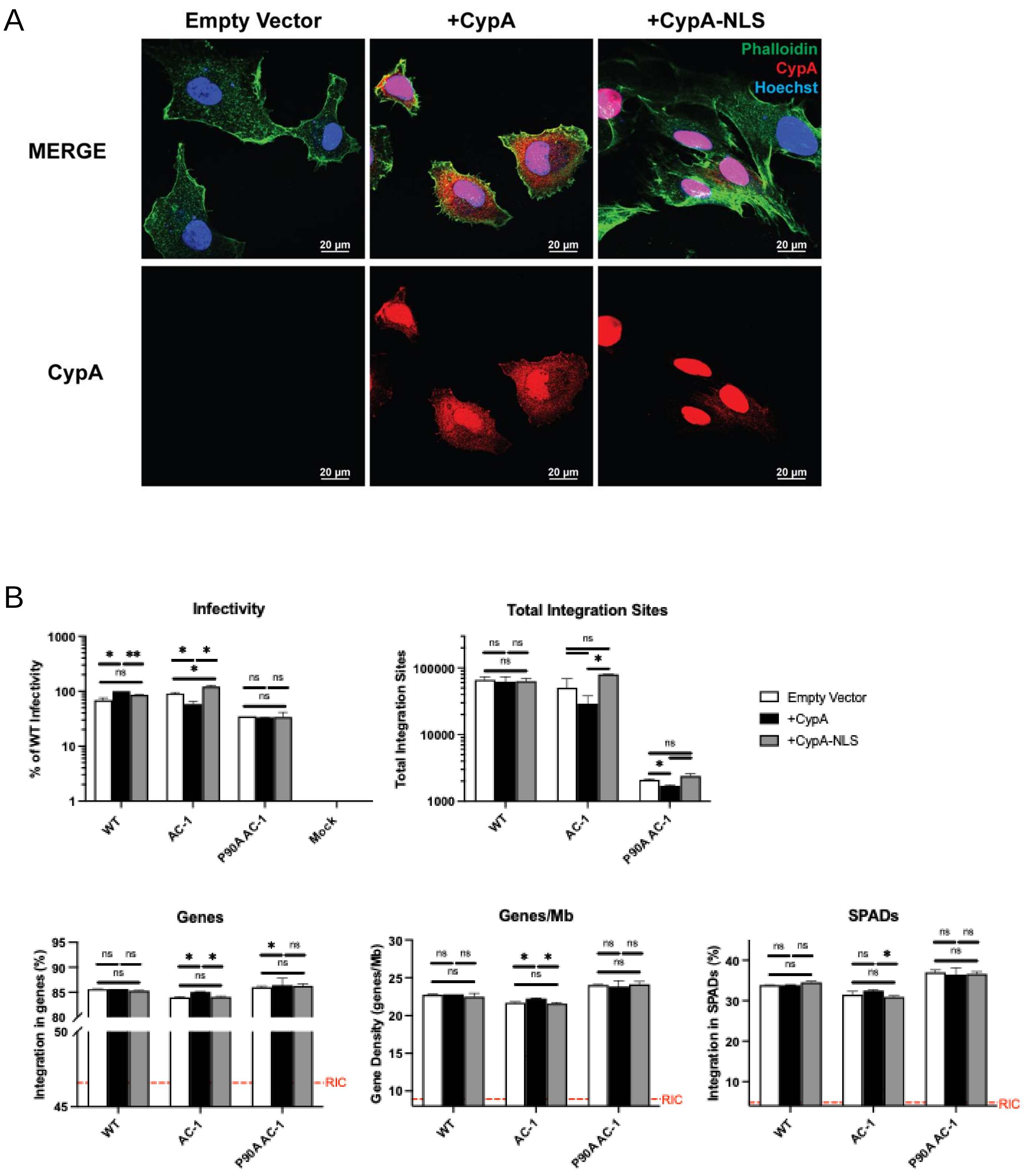
A
Empty Vector
+CypA
+CypA-NLS
MERGE
CypA
Phalloidin
CypA
Hoechst
20 µm
B
Infectivity
% of WT Infectivity
WT
AC-1
P90A AC-1
Mock
Total Integration Sites
Total Integration Sites
Empty Vector
+CypA
+CypA-NLS
Genes
Integration in genes (%)
RIC
Genes/Mb
Gene Density (genes/Mb)
SPADs
Integration in SPADs (%)
ns
*
**
